# Exposure to parks through the lens of urban mobility

**DOI:** 10.1140/epjds/s13688-022-00351-9

**Published:** 2022-07-18

**Authors:** Ariel Salgado, Ziyun Yuan, Inés Caridi, Marta C. González

**Affiliations:** 1 Instituto de Cálculo, UBA-CONICET, Ciudad de Buenos Aires, Argentina; 2 Department of Landscape Architecture and Environmental Planning, UC Berkeley, California, US; 3 Department of Civil and Environmental Engineering, UC Berkeley, California, US; 4 Department of City and Regional Planning, UC Berkeley, California, US; 5 Lawrence Berkeley National Laboratory, UC Berkeley, California, US

**Keywords:** Parks, Segregation, CDR, OSM, Census, Green spaces, Exposure, Demand, Accessibility

## Abstract

This work presents a portable framework to estimate potential park demand and park exposure through bipartite weighted networks. We use mobility information and open spatial information. Mobility information comes in the form of daily activities sampled from a model based on Call Detail Records (CDR). Spatial information comprise parks represented through OpenStreetMaps polygons and census tracts from the 2010 decennial US Census. The framework summarizes each city’s information into one bipartite weighted network with the link weights representing the number of potential visits to a park from each census tract on an average weekday.

We compare park exposure and park demand in Greater Los Angeles and Greater Boston in a pre-pandemic scenario. The park exposure of a census tract is calculated as the number of parks surrounding the daily activities of its inhabitants. The demand of a park is calculated as the number of daily activities surrounding it. We find that both cities’ distribution of park exposure have similar shape with Boston having a higher average. On the other hand, the distribution of park demand is very similar in both cities, although their park spatial distributions are different. We include racial/ethnic information from the Census to explore how the park exposure connects tracts of different racial/ethnic groups. We associate parks to racial/ethnic groups based on the number of visitors from each group. Parks within minorities’ tracts are mostly used by majority groups. Finally, through detecting communities in the network, we find that park exposure connects the cities locally, linking parks to their tracts nearby. Furthermore, we find a significant spatial correlation between network communities and different racial/ethnic composition in Los Angeles. This way, patterns of park exposure reproduce the separation among demographic groups of the city.

**Supplementary Information:**

The online version contains supplementary material available at 10.1140/epjds/s13688-022-00351-9.

## Introduction

During the COVID-19 pandemic, urban green spaces have proven to play a fundamental role as open areas where people can develop healthy habits, socialize, and obtain mental relief, among other benefits [1–4]. However, these benefits are not restricted to health emergency situations. The World Health Organization stated in 2016 that “Urban green spaces, such as parks, playgrounds, and residential greenery, can promote mental and physical health, and reduce morbidity and mortality in urban residents by providing psychological relaxation and stress alleviation, stimulating social cohesion, supporting physical activity, and reducing exposure to air pollutants, noise and excessive heat.” [5]. While green spaces have been associated with the inhabitants “happiness” [6], they tend to be left aside by governments while assessing and improving citizens’ well-being [7].

Opportunities for visiting green spaces are not equally distributed across populations and cities. The multiple differences among cities (racial/ethnic distribution, history, population, and others) result in seemingly contradictory conclusions in studies characterizing green space access and exposure for different social groups [8–10]. In some studies, minorities and low-income sectors appear to have lower access and exposure to green spaces, while in others the conversely appears to be true. This may be due to the differences in each city’s developing history. This situation applies to the US, where authors have found less trees in minorities’ neighborhoods [11], longer travel distances to reach parks for minorities [12] and poorer quality parks in lower income neighborhoods [13], but also greater access to green-ways by minorities [14] and similar access to parks by the total population [15].

Measuring the opportunities a city offers (be it green spaces or other amenities in general) presents a methodological challenge on its own. The definition of exposure varies greatly between studies, ranging from the existence of green spaces in the person’s home surroundings [16] to the actual presence of the person in a specific place [12]. Depending on the available resources, researchers may use surveys, spatial information, and mobility information, among others. Each one presents benefits and limitations. Surveys allow interacting directly with the population under study but tend to be expensive, and generally few in the number of cases considered. Spatial information alone can provide static measures that do not consider the mobility of the population, but give an idea of opportunities available. For example, in [16, 17] the authors used a greenness index based on LANDSAT satellite images, and associated green exposure to the greenness level in the surroundings of the population’s homes, finding a positive effect of the greenness on health. In [18] the authors correlate demographic indicators (such as % of Black, Hispanic or populations subsisting below the poverty line) with park presence, finding no clear association between them. In [19] park quality and proximity are shown to be correlated with their use through a combination of geographic information and surveys. Independently of the demographic group, results from [19] indicate that the determinant factor for park use is its presence nearby. In [9] the authors used an access model to measure access to green spaces in Chicago, and compared it to the racial/ethnic and economic demographic distribution. They found that census tracts with a high percentage of Hispanic and Black population had lower access to green spaces and that this situation worsened for low-income census tracts. In [20] the authors used geographic information in Los Angeles to construct several indicators of income, race and development, finding that non-White and low-income groups have much lower park access in Los Angeles. In summary, most studies on green space exposure based on spatial information alone measure exposure considering the amount of green space surrounding the locations of interest. The major difficulty they face is the definition of “surroundings”, typically using a fixed distance selected by the researcher. This distance is hard to select as it is intended to represent the region within which the population may move. Without the use of mobility information, researchers need to use ad-hoc criteria to define it. These ad-hoc criteria seem to produce incorrect estimates [12]. One possible solution to this problem is taking into account the actual behavior of the population in the analysis. This can be done by including passively collected mobility information like Call Details Records (CDR), location information from cellphone applications, or credit card usage records (see [21] for a survey on different approaches on using mobility information to measure access). Mobility information has been used to measure park exposure and park accessibility, taking into account the characteristics of the population through census information. For example, in [12], the author found a lower park exposure and a higher travel distance to parks for Black and Hispanic populations compared to White populations, in several cities in the US. In this case, park exposure was measured as the cellphone’s user presence within a park. Authors in [10] found, through the use of CDRs, that economically vulnerable groups are not susceptible to lower park access in Shanghai, China. In [22] the authors used location information from Twitter to explore the effects of park usage, finding that it had a positive effect on the emotions represented through the tweets. In [23] the authors used park exposure metrics provided by SafeGraph to measure the effect of COVID-19 policies on park usage in 44 large US cities. They found that park usage lowered compared to previous years. Moreover, after park reopening, the proportion of non-White visitors remained lower when compared with White visitors. In [24] the authors found a negative association between crime and park presence, especially when parks had a high number of visitors. They measured visits to parks using cellphone traces provided by Carto. The combination of spatial information with mobility data has been exploited for many other purposes, including estimating building occupancy [25], travel demand [26], detecting commuting patterns [27], and assessing disaster management [28], among many others. In each case, the addition of massive mobility information opens a way to include the population’s behavior. This becomes crucial when the objective is to measure the actual usage pattern, and not an expected potential value, which may differ [12].

In this work, we propose to explore the differences in park exposure in two urban areas, Greater Boston and Greater Los Angeles. These two regions present different park geographical distributions and racial/ethnic proportions across census tracts (see Materials section for a description). We follow a definition of park exposure similar to [12]. We consider that inhabitants are exposed to parks during their trajectories if there are parks near their activities (park exposure). We also define the measure from the park’s point of view (park demand) by quantifying each park’s demand as its number of potential visitors. Comparative studies among regions like [12] can help increase our understanding of the contradictory results found in the literature. Moreover, we follow a Network Science approach to assess how the park exposure connects the census tracts in each city. Park exposure can be thought to link different regions of a city, working as a connector among different demographic groups. This way, we aim to give answers to the following questions:

a How do park exposure and park demand vary between Boston and Los Angeles?
b How do parks connect different demographic groups in these cities?
c How does park exposure link different regions in these cities?

**Figure 1:**
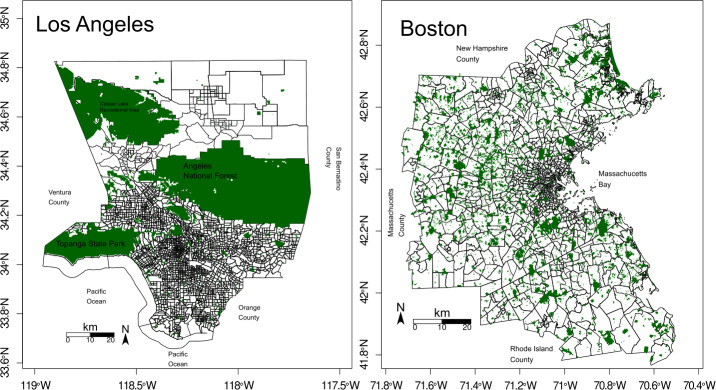
Parks in Los Angeles and Boston. The parks used for the study are presented in dark green, and census tracts are depicted in the back

We study how park exposure and demand compare in Boston and Los Angeles. Several studies, like [29], find that a uniform distribution allowing to reach a park within a short walk is critical for an equal distribution of park exposure. However, methods not including mobility information may heavily fail in predicting people’s actual behavior, as pointed in [12]. As discussed before, green space use for different demographic groups (question b) has been deeply studied, usually from the perspective of the groups. Instead of measuring how much access each group gets, we propose adding the park’s point of view, taking into account how the park relates to the places of origin of its visitors. In question c) we are interested in finding how the city connects itself through park exposure. Question c) entangles with b), as we can compare the grouping based on park exposure and based on demographic characteristics. We answer these questions by studying the bipartite network connecting parks and census tracts, with each link accounting for the number of (potential) visits from a census tract to a park. Regarding the cities under consideration, Los Angeles and Boston provide two contrasting examples, as they have a different spatial distribution of parks (see Materials section and Fig. 1). While in Los Angeles, most of the parks in the urban center are small, and the larger parks are on the suburbs, in Boston, there are parks of medium size distributed evenly throughout the region (see Materials section). On the other hand, Los Angeles has a much larger proportion of Hispanic inhabitants than Boston, where the White population is predominant in the majority of the census tracts.

The following sections present a portable framework for transforming daily mobility trajectories to a weighted network connecting a city’s census tracts and parks, applying a case study to Boston and Los Angeles in the US. While the network representation may seem excessive for simple metrics, it allows taking advantage of more complex ones, like community detection. The network representation for usage studies also provides a common ground for considering different types of usage (for example, parks and hospitals). As a case study, we use synthetically generated daily human trajectories in a typical weekday, using the TimeGeo CDR-based model [30] for Boston and Los Angeles respectively, OpenStreetMap’s park polygons from 2019 and census tracts from the 2010 Decennial Census (presented in Sect. 2). To answer question a), we measure each census tract’s park exposure and each park’s demand in terms of the number of daily activities realized in the surroundings of a park (Sect. 3.1). We compare Boston and Los Angeles through their park exposure and park demand distributions. We find that while the average park demand is similar in each city, the more even park spatial distribution of Boston favors a greater park exposure for its inhabitants. To answer question b), we label each census tract by its predominant racial/ethnic group and each park by its predominant group of visitors (Sect. 3.2). Comparing the park (tract) label with its neighboring tracts (parks) allows us to identify how different racial/ethnic groups connect through park exposure. We find that parks mostly accessed by minorities also have numerous visits from majority groups (White in Boston and White and Hispanic in Los Angeles). In contrast, the major part of minorities’ park exposure comes from parks that are predominantly accessed by the majority group. To answer question c), we explore how different regions of each city are connected through park exposure using community detection (Sect. 3.3). The obtained communities show that park usage is mainly local, linking a park to its surrounding tracts. We also found a significant spatial correlation between the detected communities and the cities’ racial/ethnic distribution, suggesting that similar demographic groups have similar park exposure patterns.

## Materials and methods

Our study uses three sources of information. The first one is spatial data representing each city’s geographical and demographic structure. It includes the census tracts and census information [31]. The second one is the parks’ geographical information, provided by the OpenStreetMaps (OSM) [32] public repository. The third one is mobility information representing daily trajectories, generated through the TimeGeo CDR-based model [30]. We combine these three data sets into one bipartite network, connecting census tracts with parks based on the population’s daily activities. In the following, we describe in detail each source of information and the method for constructing the network.

### Census spatial data: census tracts

We use the census tracts (which we call tracts for short) of Greater Los Angeles (Los Angeles for short) and Greater Boston (Boston for short) areas provided by the 2010 U.S. census. Los Angeles has about twice the number of tracts and population of Boston (see Table 1). In Los Angeles, we left out of the analysis two tracts corresponding to islands in the southernmost region of the county.

**Table 1:** Spatial and mobility data summary. Spatial data includes tracts from the Census, and parks from OSM. Mobility information includes TimeGeo trajectories and location of *other*-type activities (OA). P.A. stands for park area and U.P.A. for urban park area (parks smaller than 1 km^2^). OA with park exposure correspond to OA with parks not farther than 200 m. Trajectories with park exposure (traj. w/ park exposure) are trajectories including at least one OA with park exposure. MTD to OA stands for median of the travel distance from home to the *other* type activities (euclidean distance between *home* and *other* activities, calculated for each trajectory)

Data type	Source	Variable	L.A.	Boston
Geo spatial	Census	Population	9,814,509	4,457,728
	Census	City area (km^2^)	10,865	7317
	Census	Number of tracts	2344	975
	OSM	P.A.∥U.P.A. (km^2^)	3491∥78	1084∥502
	OSM	Parks∥Urban parks	2172∥2135	7569∥7376
Mobility	TimeGeo	trajectories	4,731,505	3,505,844
	TimeGeo	OA	4,848,350	3,294,692
	TimeGeo	MTD to OA	10.97 km	10.67 km
Both	TimeGeo + OSM	traj. w/ park exposure	695,958	1,136,720
	TimeGeo + OSM	OA w/ park exposure	751,850	1,310,857

We measure each tract’s racial and ethnic composition using the number of self-reported races and ethnicities in each census tract. We consider the proportion of White, Black, Asian and Hispanic self-reports, joining the remaining options in the category Other. We label each census tract according to the most predominant group (there weren’t tracts with Other as the most prominent category). Thus, for example, we call *Hispanic tract* a tract where the majority of the population is self-declared as Hispanic. Section S3 in the Additional file 1 shows mean proportions and number of tracts under each category. Los Angeles is one of the most diverse regions of the US [20], where we can find large regions corresponding to each demographic group. On the other hand, the White population is predominant in most of the Boston tracts. The non-White population is mostly present in the center of the city, while there are some Hispanic tracts in the North (a map presenting the racial composition of each city can be found in Figs. 7 and 8 for Boston and Los Angeles, respectively).

### OSM spatial data: parks

We use OSM to obtain the parks within each city. While OSM data is collected via crowdsourcing, it is ubiquitous, making it an appealing spatial data source [33]. Spatial objects in OSM are tagged under various categories [34], based on the uses they have. We consider a *park* every polygon with leisure tag equal to park, dog_park, playground, garden, golf_course, or with landuse tag equal to recreation_ground or nature_reserve, or natural tag equal to beach, or boundary tag equal to protected_area, following the definition used in [12]. It is worth mentioning that while park polygons provide us with valuable information regarding the shape and position of the parks, they do not include further information regarding the quality and appearance of each park. Thus, adding each park’s state into the analysis falls out of the scope of this work.

The obtained park polygons may overlap. For example, a park may include a dog_park. To consider each park only one time, we detect all the pairs of parks sharing an intersection using the sf R package. Then, we merge every intersecting pair of parks until obtaining a spatially disjoint set of polygons. When merging two polygons, the resulting polygon represents the union of the regions of each polygon. This way, if one polygon is contained within another, the resulting merge consists only of the bigger polygon. If the polygons overlap only partially, the merged polygon covers a region equal to the union of the polygons. More details can be found in Sect. S1 in the Additional file 1. The final number of parks is presented in Table 1, while the resulting parks are presented in Fig. 1. Table 1 shows the total park area in each city, and the area represented by parks smaller than 1 km^2^. The difference between Los Angeles and Boston is evident. Los Angeles has many very large parks in the North, while the rest are considerably smaller. There are also many very small parks in the West and South. In terms of park area, this is indicated by the drop from almost 3491 km^2^ of total park area to 78 km^2^ of parks smaller than 1 km ^2^, referred to as urban park area. According to [20], “Los Angeles was historically conceived as a place of low-density homes, each with its own private garden”. On the other hand, Boston presents a much more even park distribution with parks of different sizes throughout the entire region. Its total park area of 1084 km^2^ drops to 502 km^2^, when considering parks smaller than 1 km^2^. Boston has a long tradition of urban planning and inclusion of green spaces [35]. For example, many of its central highways are underground and covered with green open spaces.

**Figure 2:**
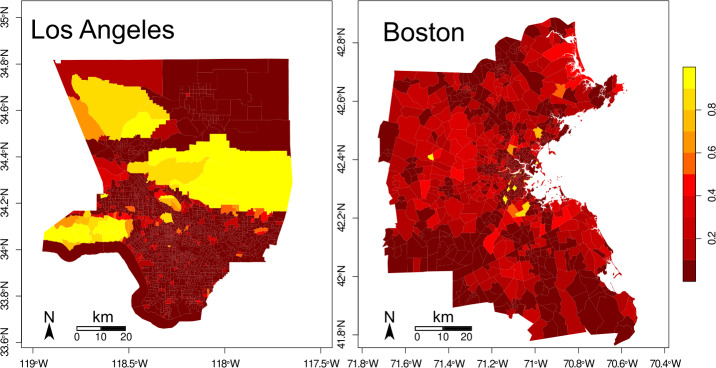
Park area proportion at each census tract. (Left) In Los Angeles, the park area is concentrated in the West and the North. Multiple parks have very small sizes and may be difficult to see at first glance. (Right) In Boston, the park area is more evenly distributed

We can get an idea of the park exposure for each tract based only on its fraction of park area (Fig. 2). For example, Boston has a total fraction of park area of 0.15, while Los Angeles has 0.28. However, considering the fraction of park area for each tract, we find that Boston has a mean park area per tract of $0.11 \pm 0.13$ while Los Angeles only has $0.04 \pm 0.12$. If we associated park exposure to the park area fraction for each tract, Boston would have, on average, almost three times more exposure, with approximately half of the total fraction of park area.

### TimeGeo trajectories for Boston and Los Angeles: mobility information

To characterize the daily mobility of each city, we use modeled trajectories based on Call Detail Records (CDRs). A CDR consists of a record of a cellphone event, either due to a call or a text message. Each CDR in the dataset contains an anonymous user ID, the geographical location of the interacting cellular tower, and the time at the instance of the cellphone activity. Thus, the spatial resolution of the localization of mobile phone users ranges from 50 m in densely populated areas to 300 m in sparse ones. The CDR data collected in Los Angeles represents six weeks in October and November 2012. For Boston, the period is two months in February and March 2010. We assume that the urban structure for one city remains almost unchanged within a few years. Thus, although the periods of the mobile phone data do not match exactly with the population and park data, it does not affect our analysis.

While CDRs offer multiple opportunities, they also may present several difficulties. Particularly, cellphone usage has been found unequal among geography, gender, and age [36]. In addition, uneven distribution in time and space of CDRs could make them prone to misrepresent the behavior of low intensity phone users [36]. The geographical and temporal sparsity may be softened by using a model capable of detecting patterns in the daily mobility of the citizens. This is the case of TimeGeo [30]. TimeGeo is a primarily CDR-based mechanistic modeling framework that generates urban mobility patterns with a resolution of 10 min and approximately 400 m, representing the minimal duration of an activity and its geographic resolution. The model uses stay locations (regions where the phone users remain a minimum lapse of time) extracted from CDRs to characterize a city’s mobility patterns. TimeGeo can generate daily trajectories of the residents of a city, consisting of several visited location points per day labeled according to the inferred activities realized in them. TimeGeo divides locations into three categories: *home*, *work*, and *other*, depending on the activity realized there. Both *home* and *work* locations are uniquely defined, meaning that each agent has only one associated *home*, and one associated *work*. However, they may transit to both of them many times during their daily trajectory. *Other* type activities are visited locations different from *home* and *work*. Agents may arrive at multiple different *other* locations during the day. The locations are selected initially from a grid over the city, with 400 m side cells representing the region where the activity occurs. We refer the reader to the TimeGeo original article [30] for a detailed explanation of the application of the model to the Boston area.

The *work* activities are dominated by commuting [30] and thus behave differently from *other* activities. For the purpose of this work, we consider only the *home* and *other* type locations, and focus on exposure to parks during *other* type activities (i.e. during non-*work* activities). Each location carries uncertainty from the 400 m side grid from which the point was sampled. We represent this uncertainty by a circle of radius $r=200$ meters around each location point. Within this circle, the agent may be located anywhere. Table 1 shows the number of *other* activities and trajectories considered, representing a typical weekday.

**Figure 3:**
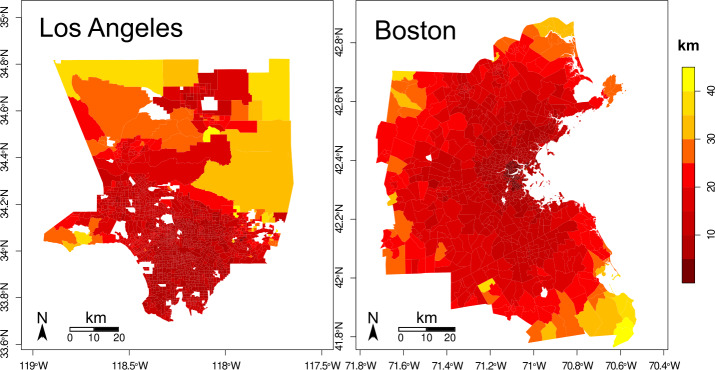
Typical travel distance from home to other activities. The color of each tract represents its median travel distance from *home* to *other* type activities. (Left) In Los Angeles, the median travel distance average value is $12 \pm 5$ km. (Right) In Boston, the average median travel distance is $13 \pm 7$ km

In Fig. 3 we present the median of the travel distance from *home* to *other* locations for each trajectory, calculated as the median of the euclidean distance between those points for each tract. We consider the euclidean distance instead of the street distance (the travel distance taking into account the streets) as we are interested only in comparing the mobility of each tract, and the euclidean distance is a simpler measure. In Boston, as we move away from the center of the city, the median travel distance increases. In Los Angeles, most of the tracts in the city’s center have a similar value, only increasing at the North and the West of the city. Boston has a median travel distance of $13 \pm 7$ km, and Los Angeles $12 \pm 5$ km. The median travel distance is similar in both cities, having a smaller standard deviation in Los Angeles.

### Network construction

The proposed framework aims to construct a bipartite weighted network for each city, representing the exposure to parks from each tract through daily activities. This network has the parks as one type of node and the tracts as the other. A link between a park and a tract represents the amount of (potential) visits from that tract to that park. Thus, we use the trajectories generated through TimeGeo to connect census tracts with parks. The trajectory $u_{a}$ of agent *a*, consists of a *home* and several *other*-type locations. We identify the census tract containing its *home* location, called $t^{a}$. The $u_{a}$ trajectory contributes to the link between the tract $t^{a}$ and a park *p* in an amount equal to the number of *other* type locations having park *p* within its uncertainty circle. Given that a park overlaps with the uncertainty circle around a location, we say that the location has exposure to the park.

**Figure 4:**
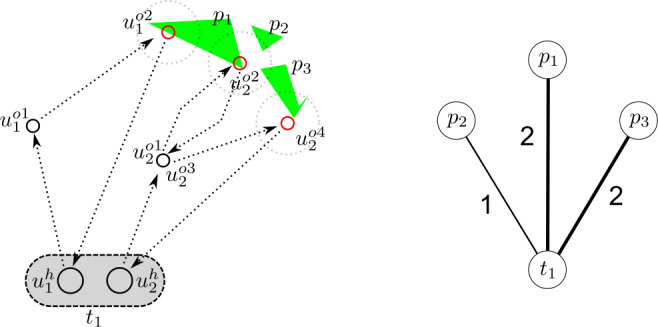
Network construction example. (Left) Trajectories $u_{1}$ and $u_{2}$ depart from tract $t_{1}$; $u_{1}$ starts at $u^{h}_{1}$, and moves through $u^{o1}_{1}$ and $u^{o2}_{1}$, the latter being exposed to park $p_{1}$; $u_{2}$ starts at home $u^{h}_{2}$, and moves through $\{u^{oi}_{2}\}^{4}_{i=1}$, with $u^{o2}_{2}$ being exposed to all the parks and $u^{o4}_{2}$ only to $p_{3}$. (Right) Resulting network with $t_{1}$ as tract node and $\{p_{i}\} ^{3}_{i=1}$ as park nodes. The weight of the links represents the number of *other* type locations exposed to each park

We see an example of how to construct the bipartite network between parks and tracts in Fig. 4. In the example, the city only has one tract $t_{1}$, and three parks $p_{1}$, $p_{2}$ and $p_{3}$. We consider two trajectories, $u_{1}$ and $u_{2}$, which share their home in $t_{1}$. Trajectory $u_{1}$ includes two *other* locations, $u^{o1}_{1}$ and $u^{o2}_{1}$, where only $u^{o2}_{1}$ is exposed to park $p_{1}$. Trajectory $u_{2}$ includes four *other* locations ($u^{o1}_{2},u^{o2}_{2},u^{o3}_{2},u^{o4}_{2}$), two of which have the same spatial location, but occur at different times ($u^{o1}_{2},u^{o3}_{2}$). $u^{o2}_{2}$ is exposed to all the parks $p_{1}$ to $p_{3}$, and $u^{o4}_{2}$ only to $p_{3}$. We construct an incidence matrix *X* with one row (one tract) and three columns (three parks), where the element $X_{ij}$ indicates the number of *other* locations exposed to park $p_{j}$ and with home within $t_{i}$. In this example, $X = [2,1,2]$.

In general, consider the set of tracts $t_{1},\dots,t_{N_{T}}$ and parks $p_{1},\dots,p_{N_{P}}$ of a city (with $N_{T}$ and $N_{P}$ the total number of tracts and parks, respectively). Trajectory $u_{a}$ associated with agent *a* consists of a *home* location $u^{h}_{a}$ and $n^{o}_{a}$ *other* locations, $u^{o1}_{a},\dots,u^{on^{o}_{a}}_{a}$. The set of parks which are intersected by the circle around $u^{oq}_{a}$ is called $P^{q}_{a}$. During the realization of the activity at $u^{oq}_{a}$, the agent is considered to be exposed to the set of parks $P^{q}_{a}$. Then, the element $X_{ij}$ is calculated as the total number of *other* type locations that have associated home within $t_{i}$, and are exposed to the park $p_{j}$:

1 $$\begin{aligned} X_{ij} = \sum^{N_{U}}_{a=1} \sum ^{n^{o}_{a}}_{q=1} I\bigl(p_{j}, P^{q}_{a}\bigr) I\bigl(t^{a}, t_{i} \bigr). \end{aligned}$$

The function $I(y,Y)$ is 1 if $y \subseteq Y$ and 0 otherwise, where *y* represents a park or a tract, and *Y* comprises a set of parks or tracts, respectively. $N_{U}$ is the total number of agents considered. Meaning that $X_{ij}$ equals the number of *other* locations with associated home in $t_{i}$ and with exposure to park $p_{j}$. Under this definition, a trajectory may account for multiple exposures to the same park. More importantly, one *other* location may indicate exposure to multiple parks at the same time.

### Network measures

We use standard metrics from network science to analyze the constructed networks. We consider node strength to measure park exposure (the tract’s view) and total potential visits or park demand (the park’s view). The link’s weight is used to derive park visitors’ ethnic/racial composition and the mean area of a tract’s potentially visited parks, and to study how the park exposure connects parks and tracts. Communities derived from the network topology are detected based on modularity optimization. We refer the interested reader to [37] for a detailed survey on complex network analysis.

#### Park exposure and potential visits

The importance of a node (park or tract) can be measured by the number of potential visits relating to it, called strength. The use of strength as a measure of importance is extensive, including ranking objects in preference networks and active users in phone call networks, as a few examples [37]. The strength of the tract $t_{i}$ is calculated as

2 $$\begin{aligned} s^{T}_{i} =\sum^{N_{P}}_{j=1} X_{ij}. \end{aligned}$$

$s^{T}_{i}$ equals the total number of parks potentially visited during *other*-type activities. We associate $s^{T}_{i}$ to the total park exposure that $t_{i}$ gets, measured as the number of potentially visited parks. The strength of park $p_{j}$ is defined as

3 $$\begin{aligned} s^{P}_{j} = \sum^{N_{T}}_{i=1} X_{ij}. \end{aligned}$$

It represents its demand, measured as the number of potential visits it receives. It equals to the number of *other*-type activities within a distance of 200 meters to the border of the park.

#### Network weighted measures

Considering a specific magnitude for tract or park nodes (like the proportion of inhabitants from a particular racial/ethnic group or the area of a park) indicated as $\alpha _{i}$ for tract $t_{i}$ or $\beta _{j}$ for park $p_{j}$, we can calculate its average value over the neighbors of a node (tract or park):

4 $$\begin{aligned} \begin{aligned}& \hat{\beta}_{i} = \frac{1}{s^{T}_{i}}\sum ^{N_{P}}_{j=1} X_{ij} \beta _{j}, \\ &\hat{\alpha}_{j} = \frac{1}{s^{P}_{j}}\sum ^{N_{T}}_{i=1} X_{ij} \alpha _{i}. \end{aligned} \end{aligned}$$

$\hat{\beta}_{i}$ is an average value assigned to a tract $t_{i}$ representing the average value of $\beta _{j}$ over the park connected to tract $t_{i}$, weighted by the fraction of potential visits to each park. For example, suppose $\beta _{j}$ represents the area of park $p_{j}$. In that case $\hat{\beta}_{i}$ represents the average area of a visited park by inhabitants of $t_{i}$, weighted by the number of potential visits to each park. $\hat{\alpha}_{j}$ represents the average value of $\alpha _{i}$ over the tracts neighboring park $p_{j}$. For example, if $\alpha _{i}$ represents the fraction of White inhabitants of tract $t_{i}$, then $\hat{\alpha}_{j}$ is the average fraction of White visitors of park $p_{j}$.

#### Parks and tracts homophily

Given a set of categories over tracts and parks (for example, a label indicating the predominant race/ethnic group of its inhabitants or visitors), we can further inspect the category combinations associated with their links. Assume we have the categorical labels $\{g^{T}_{i}\}^{N_{T}}_{i=1}$ for the tracts and $\{g^{P}_{j}\}^{N_{P}}_{j=1}$ for the parks. The homophily of a node (park or tract) represents its similarity with its network neighbors. Homophily is used to identify how the network mixes the categories of its nodes. Some applications are the mixing of male-female dolphins in dolphin social networks, race mixing in partnership networks and connectivity patterns between providers and users in Internet networks [37]. It is defined as the fraction of a node’s neighboring nodes sharing its label:

5 $$\begin{aligned} \begin{aligned}& h^{T}_{i}= \frac{1}{s^{T}_{i}} \sum^{N_{P}}_{j=1} X_{ij} \delta _{g^{T}_{i},g^{P}_{j}}, \\ &h^{P}_{j}= \frac{1}{s^{P}_{j}} \sum ^{N_{T}}_{i=1} X_{ij} \delta _{g^{T}_{i},g^{P}_{j}}, \end{aligned} \end{aligned}$$

where $\delta _{g^{T}_{i},g^{P}_{j}}$ equals 1 only if both regions have the same label and 0 in other cases. $h^{T}_{i}$ ($h^{P}_{j}$) represents the fraction of neighboring parks (tracts) with the same label of tract $t_{i}$ (park $p_{j}$). A value of $h^{T}_{i}$ near 0 indicates that inhabitants of tract $t_{i}$ mostly visit parks which are mostly visited by other racial/ethnic groups. A value of $h^{T}_{i}$ near 1 indicates that inhabitants of $t_{i}$ mostly visit parks which are mostly visited by the predominant racial/ethnic group of $t_{i}$.

#### Communities

The network’s structure can be used to detect heavily connected subgroups of nodes, called communities. Community detection is one of the most intensively researched areas in Network Science. Applications include racial mixing in friendship networks, topic analysis in coauthorship networks, and identification of functional units in software networks [37]. In our case, a community can associate groups of parks and tracts showing common use of parks and a tendency to visit the same places. To quantify how strongly connected a set of nodes is, the most common approach is to use the modularity, defined for bipartite networks as [38]:

6 $$\begin{aligned} Q = \frac{1}{m} \sum^{N_{T}}_{i=1}\sum ^{N_{P}}_{j=1} \biggl(X_{ij}- \frac{s^{T}_{i} s^{P}_{j}}{m}\biggr) \delta _{c^{T}_{i},c^{P}_{j}}, \end{aligned}$$

where $c^{T}_{i}$ and $c^{P}_{j}$ indicate to which community $t_{i}$ and $p_{j}$ belong, respectively. $m = \sum^{N_{T}}_{i=1}\sum^{N_{P}}_{j=1} X_{ij}$ is the total weight of the network. $s^{T}_{i} s^{P}_{j}/m$ represents the expected number of potential visits from $t_{i}$ to $p_{j}$ if potential visits were equally distributed between every park and tract, and *m* represents the total number potential visits to parks considered. We use the leading eigenvector method [39] to detect the partitions with the higher modularity.

## Results

Table 2 shows the final number of nodes of each type in the network (number of parks $N_{P}$ and number of tracts $N_{T}$), the number of connections *L*, and the total weight *m* (representing the total number of potential visits to parks) of each network. Boston has nearly three times as many parks as Los Angeles, but less than half the tracts. The number of links *L* in Boston is around twice that of Los Angeles’. Boston’s average tract connects to $L/N_{P} \sim 650$ parks and Los Angeles’ average tract connects to ∼170 parks, representing $\sim 10\%$ of the total number of parks in Boston and $\sim 8\%$ in Los Angeles. The average number of potential daily visits to a park from a tract is $m/L \sim 4$ in Boston, while ∼2.5 in Los Angeles. The average park demand $m/N_{P}$ (the number of potential daily visits a park receives) is 422 in Boston, with a similar value of 472 in Los Angeles. Boston’s average park exposure, $m/N_{T}$, accounts for 2628 parks, while Los Angeles’ average park exposure is only 424, six times less than Boston. It’s interesting that both cities have a very similar average number of potential visits $m/N_{P}$ (the park view), while the average of park exposure (the tract view) is very different. This means that an average *other* location has more parks in its vicinity in Boston than in Los Angeles, while an average park is surrounded by a similar number of *other* locations in both cities. While the average park has a similar demand on both cities, inhabitants from Boston have more parks in the vicinity of their activities, resulting in a higher exposure to parks.

**Table 2:** Summary network information. Final number of parks $N_{P}$, final number of tracts $N_{T}$, number of links *L*, total weight *m* and average weight $m/L$ for the constructed networks

	Boston	L.A.
Parks	5940	2026
Tracts	956	2256
Links	622,064	386,015
Total weight	2,512,088	956,906
Average weight	4.03	2.48

### Comparing park exposure and park demand in Los Angeles and Boston

To explore how park exposure changes from one city to the other (question a), we calculate how park exposure (Eq. (2)) and park demand (Eq. (3)) are distributed in each city. Later, we consider how the area of the parks relates to the exposure, calculating each tract’s average exposed park area.

**Figure 5:**
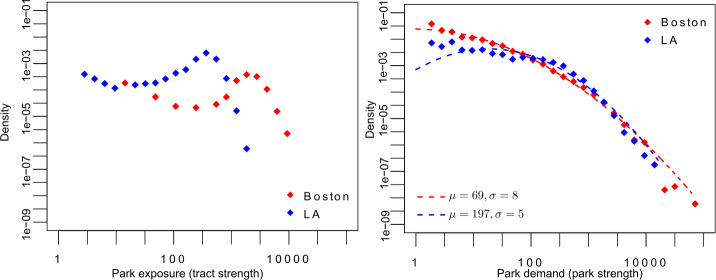
Park exposure and park demand. (Left) Histogram of park exposure for each city. (Right) Distribution of park demand. Points represent density of cases, on a log-binned histogram. The distributions are reasonably represented by log-normal distribution (dashed lines) with mean *μ* and standard deviation *σ* (both in natural scale). In both panels, points represent density of cases, on a log-binned histogram

Figure 5 shows in the left panel the distribution of park exposure for both cities. As discussed before, both cities’ distributions are similar but centered on different values. Boston’s tracts have higher park exposure than Los Angeles’ tracts on average, in accordance with its bigger mean fraction of park area per tract and broader distribution of median travel distance.

Looking at the park demand distribution in the right panel of Fig. 5, we see almost no difference between cities. In this case, log-normal distributions represent both distributions well. Although the distributions have differences at their lower values, both are very similar in most of their range. Based on the two studied cities, this indicates that from the parks’ perspective, their demand does not depend on the city or the presence of other parks, but only on the number of trajectories considered.

#### Considering the area of the parks

**Figure 6:**
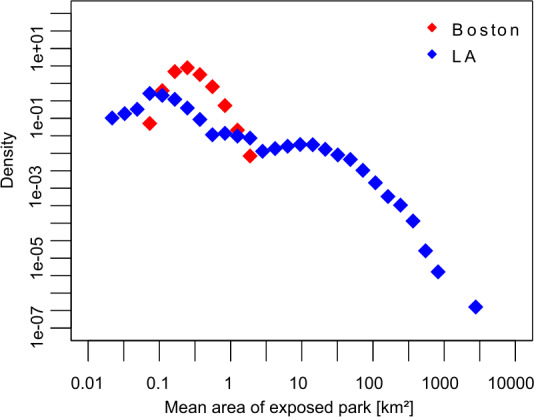
Mean area of visited parks. Distribution of the average area of the potentially visited parks for each tract. Points represent density of cases, on a log-binned histogram

Figure 5 presents the observed distribution of park exposure. However, it does not inform us on the characteristics of the visited parks. A tract’s inhabitants may visit small parks several times while others may visit large parks a few times. While the former will have a higher park exposure than the latter, the effect of this exposure may be greater for the latter. Fig S2 in the Additional file 1 shows the distribution of the parks’ area in each city. Both cities have a very similar distribution of park areas, despite having a very different spatial distribution (as seen in Fig. 1). We use Eq. (4) to calculate the average area of a potentially visited park for each tract. Figure 6 shows the distribution of the average area of a potentially visited park. It highlights an interesting difference between the cities: while the distribution of the parks’ areas is very similar in both cities, what people find regarding parks during their daily activities can be very different. This is understandable as the park’s spatial distribution is very different. In the case of Boston, the distribution has one mode at ∼0.25 km^2^. In the case of Los Angeles, we can see two modes, one at ∼0.07 km^2^ and the other at ∼9.50 km^2^.

### Racial/ethnic distribution and park exposure

**Figure 7:**
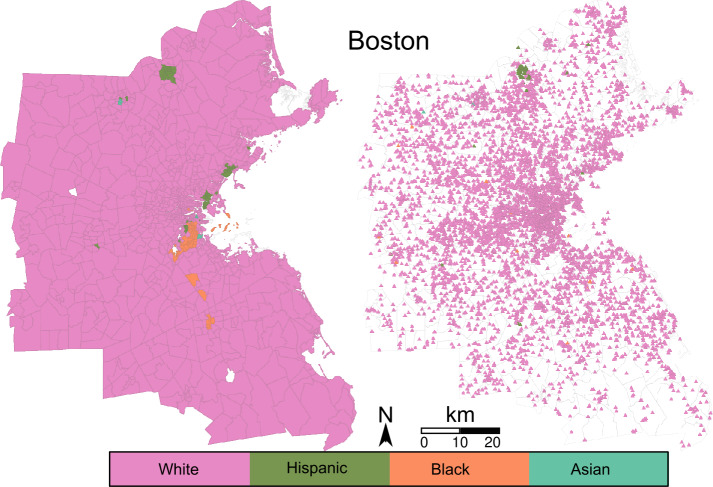
Park and tract racial/ethnic groups in Boston. Tracts are presented in the left, park centroids in the right

**Figure 8:**
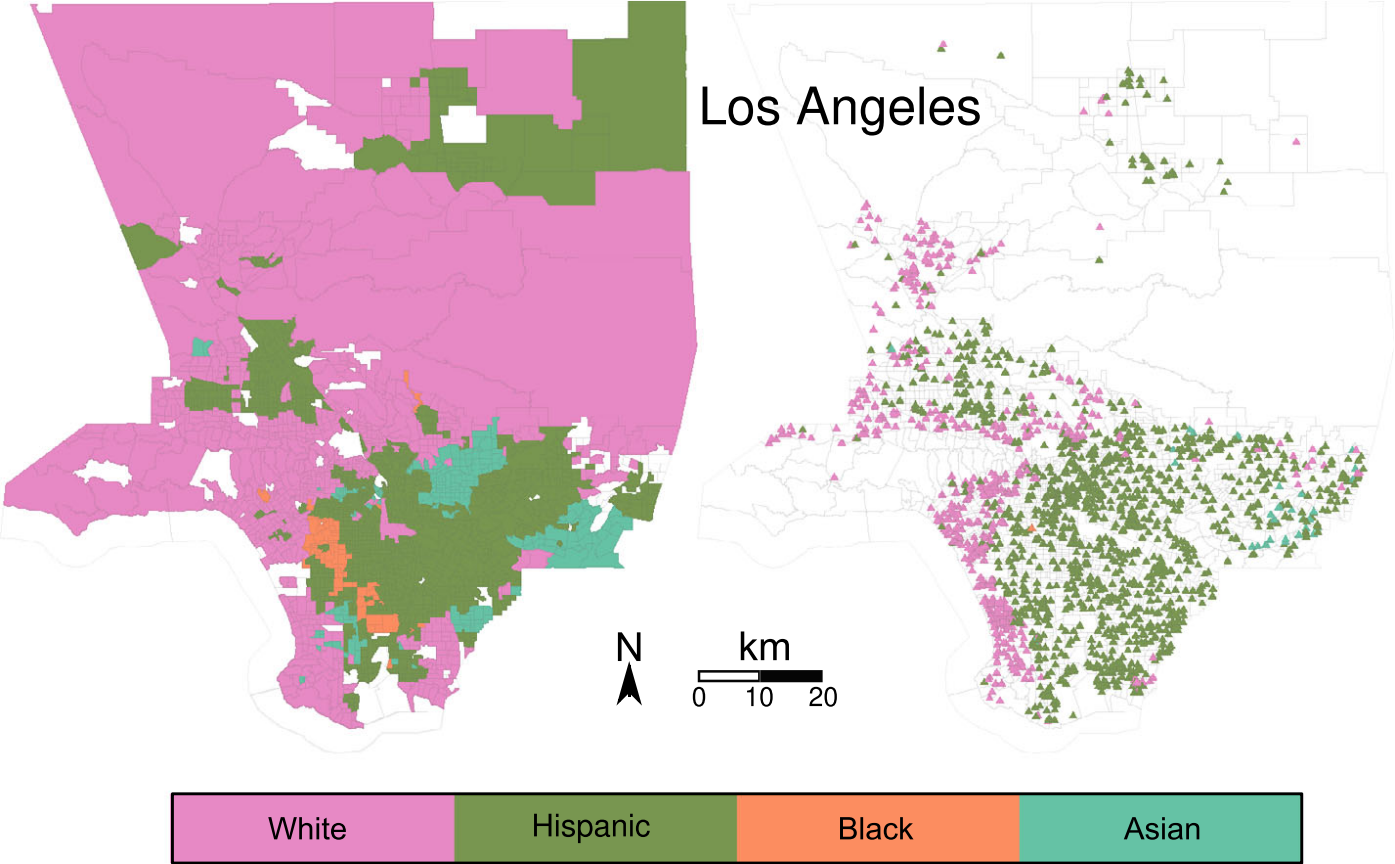
Park and tract ethnic and racial groups in Los Angeles. Tracts are presented in the left, park centroids in the right

To explore how the different ethnic/racial groups connect through park exposure (question b), we first assign each park an estimated proportion of potential users from each group, based on the composition of their visitors’ home tracts. We calculated the average proportion of White, Black, Asian and Hispanic visitors using Eq. (4) and the proportion of the population of each group in each tract. The average proportion of visitors from each racial/ethnic group is presented in Table S3 in the Additional file 1. Hence, each park and tract is labeled, indicating the predominant group who visits it (park) or who lives in it (tract). For example, we call a *Hispanic tract* a tract with a majority of Hispanic population, and a *Hispanic park* a park with a majority of Hispanic visitors. Figures 7 and 8 show the tracts and parks labeled using their predominant group for Boston and Los Angeles, respectively. White tracts are predominant in Boston, followed by Hispanic and Black tracts, and Asian tracts at last. In Los Angeles, Hispanic tracts are predominant, White tracts follow closely, and Asian and Black tracts are less. These results roughly correspond with the number of parks from each group and the percentage of the area they represent, as presented in Table 3. To explore how parks are shared among groups, we measure the fraction of parks of each group within each set of tracts. Tables 4 and 5 show these fractions. Notoriously, in Boston the few Asian and Black parks are located within White tracts. Only the 22% of parks within Hispanic tracts are Hispanic parks, and the rest are White parks. In Los Angeles the Hispanic group is predominant. In contrast with Boston, Asian and Black groups have parks with majority of their own group within their tracts. However, Hispanic parks are predominant in Asian and Black tracts. Thus, we see that the majority group populates the major part of the parks of each city.

**Table 3:** Number of parks and tracts from each group. We calculate the number of tracts from each group, the percentage of area of the city they represent, the number of parks from each group, and the percentage of the total park area they represent

	Boston				Los Angeles			
	Tracts	% Area Tracts	Parks	% Area parks	Tracts	% Area Tracts	Parks	% Area parks
Asian	6	0.1	2	0.003	189	3.5	47	0.02
Black	55	0.7	14	0.032	74	1.1	1	5 × 10^−5^
Hispanic	58	0.5	48	0.133	1260	23.4	1322	84.7
White	837	98.7	5876	99.833	731	72.0	632	15.3

**Table 4:** Parks within different racial/ethnic regions in Boston. For each racial/ethnic group, we consider its set of tracts. We compute the fraction of parks from each group, and the total number of parks in the region

	Parks				
Tracts	Asian (%)	Black (%)	Hispanic (%)	White (%)	Total
Asian	0	0	0	100	13
Black	0	0	0	100	122
Hispanic	0	0	21.89	78.11	169
White	0.04	0.23	0.20	99.54	5626

**Table 5:** Parks within different racial/ethnic regions in Los Angeles, For each racial/ethnic group, we consider its set of tracts. We compute the fraction of parks from each group, and the total number of parks in the region

	Parks				
Tracts	Asian (%)	Black (%)	Hispanic (%)	White (%)	Total
Asian	16.59	0	79.02	4.39	205
Black	0	2.17	89.13	8.70	46
Hispanic	0.13	0	95.70	4.18	790
White	0.55	0	36.89	62.56	908

We propose a complementary measure using the network topology instead of the geospatial topology. For this purpose, we measure group homophily for each park and tract following Eq. (5). This metric calculates the fraction of parks (tracts) that are connected to a tract (park) and have the same racial/ethnic label. For example, if a Hispanic tract has homophily equal to 1, its population only goes to parks where the majority of visitors are Hispanic. On the other hand, if a Hispanic tract has homophily equal to 0, its population only visits parks where Hispanic visitors are not majority. The reasoning for a park is similar. This measure is complementary to the results presented in Tables 4 and 5 as it tells us from where the different groups obtain park exposure, and also from which group a park attracts visitors. Table 6 presents the average homophily for each ethnic/racial group, node type (park or tract), and each city. We compare both cities from two points of view: tracts’ and parks’, and disaggregate them by group.

**Table 6:** Homophily for each group. For each ethnic/racial group and city, we present mean and standard deviation of tract homophily and park homophily (see the main text for definition). Number of parks and tracts in each group are also included

	Tract homophily		Park homophily	
	Boston	L.A.	Boston	L.A.
Asian	(2 ± 4)×10^−4^	0.02 ± 0.05	1	0.74 ± 0.21
Black	(3 ± 1)×10^−4^	(1 ± 7)×10^−3^	0.86 ± 0.21	0.49
Hispanic	0.07 ± 0.11	0.87 ± 0.11	0.68 ± 0.16	0.64 ± 0.15
White	0.99 ± 0.01	0.38 ± 0.20	0.94 ± 0.09	0.65 ± 0.12

From the tracts perspective, Boston can be separated in White (majority) and non-White (minorities) tracts. The homophily of White tracts is almost 1, indicating that the population from those tracts is only exposed to White parks. On the contrary, non-White tracts have very low homophily. This relates to the previous results, as parks within Black and Asian tracts are White parks. Thus, the major part of the exposure of these groups occurs in parks predominantly visited by White inhabitants. The $\approx 22\%$ of Hispanic parks within Hispanic tracts does not increase their homophily over 0.1. Los Angeles presents a similar situation. Asian and Black tracts almost only connect to Hispanic parks. In Los Angeles, White tracts’ homophily is below 0.5, as they connect to multiple Hispanic parks. Hispanic tracts are similar to White tracts in Boston, connecting almost only to Hispanic parks.

Parks tend to connect mostly to their own group. Boston’s parks have an average homophily of more than 0.5 in all groups. Particularly, the Asian parks only connect to Asian tracts. This indicates that a park is Asian if it only receives visits from Asian tracts’ population. White and Black parks have high homophily too. Hispanic parks have the lowest homophily, being connected to multiple White tracts. Los Angeles’ parks have homophily closer to 0.5 compared to its tracts. White and Hispanic parks have homophily around 0.65, indicating that (in contrast with Boston) they connect tracts from different groups. The only park from the Black group has homophily of 0.49, connecting evenly to Black and non-Black tracts. While they have the higher average homophily, Asian parks also connect to non-Asian tracts. In general, Los Angeles’ parks have lower homophily than Boston’s. This indicates that parks in Boston are more linked to their predominant group of visitors than Los Angeles parks are.

Comparing ethnic/racial groups across cities, we see that the predominant group of each city is also predominant in the majority of the parks. This is the case for the White group in Boston and the Hispanic group in Los Angeles. The racial/ethnic minorities are minorities in parks too. Even parks within the minorities’ tracts are mostly exposed to visitors from the majority group. This is the case for Black and Asian groups in both cities. White and Hispanic groups interchange their role from Boston to Los Angeles.

### How park exposure connects the cities?

Now we focus on analyzing how the exposure to parks connects the different regions of the city (question c). Inhabitants exposed to similar parks are prone to encounter inside them and to be exposed to similar situations. This may link regions far from each other by sharing the parks they connect to. On the other hand, from the park view, parks sharing similar visitors can work as modular units, and be seen as park complexes.

**Figure 9:**
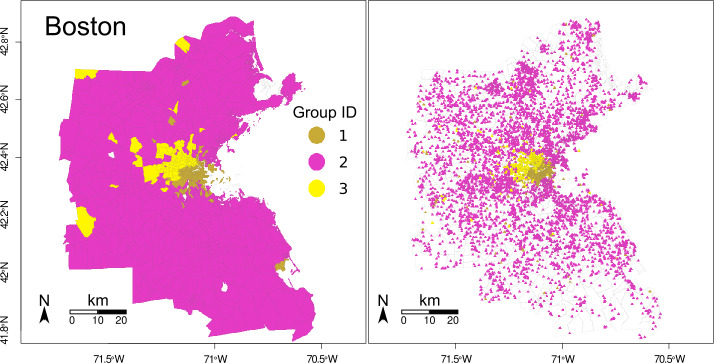
Park and tract communities in Boston. Group ID indicates the community number of each tract. Tracts are presented in the left, park centroids in the right

**Figure 10:**
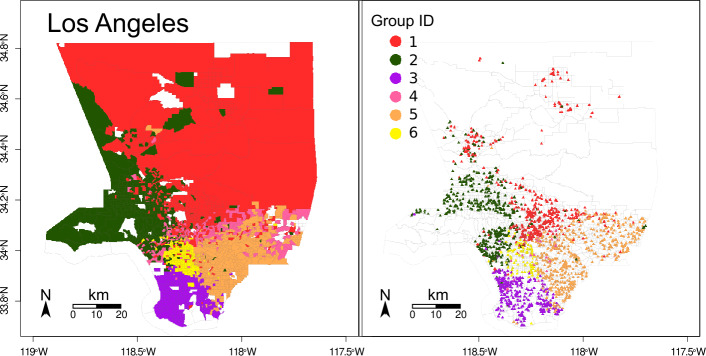
Park and tract communities in Los Angeles. Group ID indicates the community number of each tract. Tracts are presented in the left, park centroids in the right

To detect communities of parks and tracts, we use the network modularity, as defined in Eq. (6). We obtain the community structures presented in Fig. 9 for Boston (modularity of 0.16) and in Fig. 10 for Los Angeles (modularity of 0.29). As expected, the community detection method groups tracts and parks which are closer in space.

Communities found in Boston split the city into three regions, representing the downtown (1, gold), the suburbs (2, magenta), and an intermediate region (3, yellow). It is interesting that park exposure separates the center of the region (community 1) from the suburbs (community 2) which are connected in one large community. Also, while some tracts in the suburbs are more connected to the downtown and the intermediate region, the conversely is not true: parks in the downtown are used by inhabitants from the downtown.

Six communities are found in Los Angeles (Fig. 10). They represent the large parks in the North (1, red), West parks (2, dark green), the beach (3, violet), an intermediate region (4, pink), East center (5, orange) and the center of the city (6, yellow). Similar to Boston, communities group tracts from adjacent geographical regions.

Interestingly, the communities obtained by network usage and the categorization based on racial/ethnic groups seem to represent similar regions. To measure to what extent this is true, we calculated the mutual information between the racial/ethnic label and the community label. For the tracts, we obtain a mutual information of 0.031 bits (i.e., using log_2_) for Boston and 0.192 bits for Los Angeles. To compare these values with a reference, we randomly mixed the same set of community labels 5000 times (and kept fixed the racial/ethnic labels), and calculated the mutual information for each sample, obtaining $0.005 \pm 0.002$ bits for Boston and $0.005 \pm 0.002$ bits for Los Angeles. As Boston only has 3 communities, and one comprises the majority of the tracts, this is less surprising. On the other hand, the communities found in Los Angeles have a mutual information more than 100 times higher than expected by chance. Using the racial/ethnic labeling for the parks presented before, and the park’s community assignment, results in a mutual information of 0.0012 bits for Boston and 0.230 bits for Los Angeles. By mixing the community labels as done with the tracts, the mutual information results of $0.0007 \pm 0.0004$ bits for Boston and $0.005 \pm 0.002$ bits for Los Angeles. In this case, the observed value for Boston is within two standard deviations, while for Los Angeles, it is many deviations above the average situation observed by chance. This indicates that the grouping induced by mobility patterns and park exposure captures differences in the demographic population for Los Angeles, while for Boston it is only significant for the tracts.

## Discussion

We use a simple characterization of the networks, using the number of tracts, parks, links, and the links’ weight (Table 2) and observe that Boston has much more exposure to parks than Los Angeles (question a). Los Angeles, which has about twice as many tracts and population of Boston, has six times less urban park area than Boston, resulting in half the number of activities with park exposure. The even spatial distribution of parks of Boston makes parks closer to the home tracts and increases the park exposure. However, it is interesting that the park demand distribution is unaffected by the park spatial distribution (notice that both cities are comparable as we have extracted a similar number of trajectories, Table 1). Considering the typical area of a visited park separates Los Angeles’ tracts into two groups, associated with each mode in Fig. 6. The first mode corresponds to small parks, and is associated with the tracts in the city center, while the second is associated with the tracts in the North and West, surrounding the national parks. In contrast, Boston presents a unimodal distribution of the area of visited parks, meaning that there is one typical view for the whole city. This difference contrasts with the similar park area distribution of both cities (Fig S2 in the Additional file 1).

The communities induced by park exposure separate the city into geographically connected regions (question c). The mutual information between these regions and the racial/ethnic partition is higher than expected by chance. This tells us that there is a relation between which parks are visited, and the racial/ethnic characteristics of the visitors (question b). In Boston, the White population is the majority and thus the majority of parks are associated with them. On the other hand, White and Hispanic population have a comparable presence in Los Angeles. While most of the area is associated with White tracts, the majority of the parks are associated with the Hispanic population. This is understandable as Hispanic tracts are more numerous, and each census tract has a similar population.

The homophily of a tract indicates the fraction of its park neighbors in the bipartite network with its own label. Similarly, the homophily of a park indicates the fraction of its tract neighbors in the bipartite network with its own label. Tracts associated with minorities in both cities are mostly exposed to parks from the dominant group (or groups). This is a consequence of the low number of parks labelled after minorities. Spatial identification of parks from minority groups with high homophily, shows that they are labelled after minorities because they fall outside the reach of the majority group. Comparing the two regions, Los Angeles appears as a more diverse city, with lower values of park homophily, suggesting that the parks work as connectors between tracts from different groups.

## Conclusions

We presented a method to analyze park demand and park exposure of a city’s inhabitants. It uses census data, OSM park polygons, and mobility information. We analyzed Greater Boston and Greater Los Angeles areas, finding that the larger park area in Los Angeles does not imply a greater park exposure. With a more even spatial distribution, Boston obtains a higher average park exposure. Notoriously, park demand is similar in both cities, suggesting that park demand does not depend on parks’ spatial distribution. Exploring the park point of view in other cities presents an interesting research topic in the study of park usage.

Our analysis finds potential differences between racial/ethnic groups in terms of park exposure and park demand. It is interesting that parks appear as connectors between different groups. Further research using mobility information with greater spatial resolution could be conducted to assess to which extent housing racial/ethnic segregation extends to daily activities. For example, it is not clear what represents for the inhabitants of Black and Asian tracts that other communities are the predominant visitors of the parks within their tracts. In addition, including other dimensions to the analysis, such as the characteristics of the parks or their appearance, may help to disclose other aspects of exposure while still working at the urban scale.

## Supplementary Information

Below is the link to the electronic supplementary material.

Supplementary information (PDF 233 kB)

## Data Availability

Mobility information cannot be shared due to privacy restrictions. Census tracts and demographic information is directly available form the US Census Bureau web-page (https://www.census.gov/data.html). Parks are available from OpenStreetMaps (https://www.openstreetmap.org/) and can be downloaded using the tags presented in the text. The spatial data and the networks generated and analysed during the current study are available from the corresponding author on reasonable request.
