# Supplementary material for: Exposure to parks through the lens of urban mobility
Source: EPJ Data Sci. 2022 Jul 18;11(1):42. doi: 10.1140/epjds/s13688-022-00351-9 (PMC9289662; doi:10.1140/epjds/s13688-022-00351-9)
Supplement: Supplementary file 1 — Supplementary information (PDF 233 kB) [file 13688_2022_351_MOESM1_ESM.pdf]

Exposure to parks through the lens of urban mobility

Ariel Salgado Ziyun Yuan Inés Caridi Marta Gonzalez

Supplementary Material

S1 **OpenStreetMaps parks processing** After downloading the parks under the tags indicated in the Materials section, a pre-processing is needed. Many of these polygons may overlap, as portions of a park may have different detailed uses (for example, a big polygon tagged as `park` may contain smaller ones tagged as `dog_park` or `playground`). However, as we are interested in counting the number of parks in a specific region, having duplicated informat would lead to overestimating the number of parks in that region. For this reason, we loop over the parks and for each polygon we detect all the polygons intersecting it. We merge every intersecting polygon, obtaining a new multi-tag polygon, combining the tags of the merged polygons. We repeat this process until all the polygons are disjoint, using the R package `sf`. The combined result compressed the number to a 77% of the original number of parks in Los Angeles and a 69% in Boston.

To have an idea of OSM's quality, we cross-checked the parks with the park data provided by each city's government in the metropolitan area. The objective was to see to what extent OSM was representative of what was present in the government datasets. To measure the overlap, we used the Jaccard coefficient, calculated as the area of intersection of polygons over the size of the union of polygons, between OSM parks and government parks. We restrict the calculation to the bounding box containing the governments' parks (Los Angeles and little Boston). In both cities the Jaccard Coefficient was around 60%. Further inspecting we found that many regions were left outside by both parties. Taking into account the tags of the OSM parks polygons, we checked which categories had lower Jaccard coefficient, and decided to remove parks only tagged as `dog_park`, `garden` or `golf_course` after merging. The remaining parks represent 54% of the original number in Los Angeles and 60% in Boston. Table S1 Table provides a summary of these results.

S1 Table Summary information of the park data downloaded using OpenStreetMaps. Overlap and correlation is measured using the Jaccard coefficient.

	L.A.	Boston
Original‖merged‖final	4004‖3074‖2172	11731‖8113‖7084
Overlap with gov. data	61.7%	61.2%
Low Jaccard tags	garden, golf_course	beach, dog_park, garden

S2 **Distribution of park size in each city** Both cities have a very similar distribution of park area, while Los Angeles has less parks in the range 3 to 100 square meters and more over 100 square kilometers.

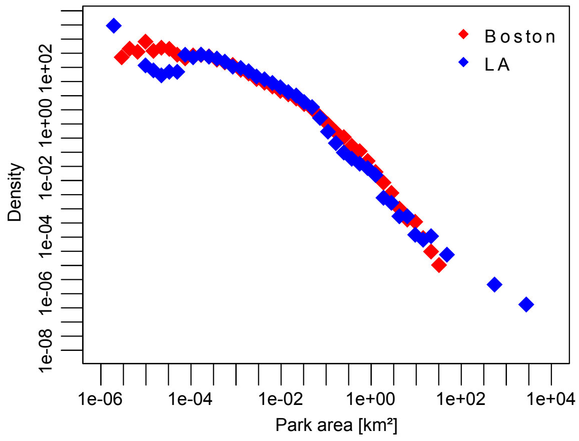

S3 **Park potential visitors' racial/ethnic proportion.** To calculate the proportion of visitors from each racial/ethnic group (g), we consider the proportion inhabitants from each racial/ethnic group at each tract t_i, α_{gi}. Using the incidence matrix X, the proportion of visitors from group g at park p_j, β_j, is calculated as:

$$\beta_j = \frac{1}{s_j^P} \sum_{i=1}^{N_T} \alpha_i X_{ij}$$

S3A Table shows mean values of α_{gi} and β_{gj} for Los Angeles and S3B Table for Boston.

S3A Table Mean values of racial/ethnic proportion in Los Angeles for parks and tracts.

g	α_{gi}	β_{gi}
White	0.28 ± 0.26	0.31 ± 0.14
Black	0.09 ± 0.13	0.08 ± 0.05
Asian	0.14 ± 0.15	0.15 ± 0.08
Hispanic	0.48 ± 0.29	0.45 ± 0.14
Other	0.01 ± 0.01	0.008 ± 0.003

S3B Table Mean values of racial/ethnic proportion in Boston for parks and tracts.

g	α_{gi}	β_{gi}
White	0.72 ± 0.25	0.79 ± 0.12
Black	0.08 ± 0.14	0.05 ± 0.05
Asian	0.07 ± 0.08	0.06 ± 0.04
Hispanic	0.11 ± 0.15	0.08 ± 0.07
Other	0.02 ± 0.03	0.013 ± 0.0123

S4 Glossary: List of variables and notations

u_a Daily trajectory corresponding to agent a.

u_a^h *home* location corresponding to trajectory u_a.

u_a^{oq} q-est *other*-type location in trajectory u_a.

n_a^o Number of *other*-type locations in trajectory u_a.

t^a home region associated to trajectory u_a

H Set of tracts

P Set of parks.

t_i The i-est census tract.

p_j The j-est park.

P_a^q Set of parks to which activity u_a^{oq} was exposed.

N_P Number of parks of the city.

N_T Number of tracts of the city.

X_{ij} Element of the incidence matrix, with $0 < i \leq N_t, 0 < j \leq N_p$. i addresses the tract, and j the park. X_{ij} represents the daily number of other locations exposed to park j whose trajectories have a home at tract i.

m Total weight of the network, calculated as $\sum_{i=1}^{N_T}\sum_{j=1}^{N_P} X_{ij}$

s_i^T Strength of tract t_i, defined as $\sum_{j=1}^{N_P} X_{ij}$. Accounts for the daily exposures to parks for trajectories with *home* at tract i. Represents the total park exposure from tract i.

s_j^P Strength of park p_j, defined as $\sum_{i=1}^{N_T} X_{ij}$. Accounts for the daily number of other activities with exposure to park j. Represents the number of potential visits to park p_j, and also its demand.

α_i Real-valued amount for t_i, for example, median income.

β_j Real-valued amount for p_j, related to α_i through $\beta_j = \frac{1}{s_j^P}\sum_{i=1}^{N_T} \alpha_i X_{ij}$.

g_i^T, g_j^P groups for tracts and parks, respectively.

c_i^T, c_j^P communities for tracts and parks, respectively.

h_i^T, h_j^P homophily values for tracts and parks, respectively..

Q Barber modularity, defined as $Q = \frac{1}{m}\sum_{i=1,j=1}^{N_t,N_p}(X_{ij} - \frac{s_i^t s_j^p}{m})\delta_{c_i^T, c_j^P}$, where c_i^T, c_j^P are the communities to which tract i and park j belong.

s_i^{TM} Modeled strength of tract t_i under model M.

M_i Model i of tract strength

S_{OL} The expected number of *other* activities per daily trajectory, regardless of their location.

$R(d,t), R_i$ Region surrounding a tract, based on a distance d to the centroid of tract t. The region consists of all tracts whose centroid distance to tract t centroid is less or equal to d. Subindex i refers to tract t_i

$f_P(R,l)$ Probability of an other activity falling inside a cell containing a park, based on region R and cell size l. It's estimated as the fraction of cells with size l in region R intersecting a park.

$k_P(R,l)$ Expected number of parks per cell, given the cell contains at least a park. It depends on region R and cell size l.

d_i Median travel distance. Subindex i refers to tract i
